# Estimation of minimum foot clearance using a single foot-mounted inertial sensor and personalized foot geometry scan

**DOI:** 10.1038/s41598-024-63124-6

**Published:** 2024-06-13

**Authors:** Katherine Heidi Fehr, Jennifer Nicole Bartloff, Yisen Wang, Scott Hetzel, Peter G. Adamczyk

**Affiliations:** 1 Mechanical Engineering Department, University of Wisconsin-Madison, Madison, WI USA; 2 Department of Biostatistics and Medical Informatics, University of Wisconsin-Madison, Madison, WI USA

**Keywords:** Translational research, Biomedical engineering, Mechanical engineering

## Abstract

The real-world measurement of minimum foot clearance (mFC) during the swing phase of gait is critical in efforts to understand and reduce the risk of trip-and-fall incidents in populations with gait impairments. Past research has focused on measuring clearance of a single point on a person’s foot, typically the toe—however, this may overestimate mFC and may even be the wrong region of the foot in cases of gait impairments or interventions. In this work, we present a novel method to reconstruct the swing-phase trajectory of an arbitrary number of points on a person’s shoe and estimate the instantaneous height and location of *whole-foot* mFC. This is achieved using a single foot-mounted inertial sensor and personalized shoe geometry scan, assuming a rigid-body IMU-shoe system. This combination allows collection and analysis using out-of-lab tests, potentially including clinical environments. Validation of single marker location using the proposed method vs. motion capture showed height errors with bias less than 0.05 mm, and 95% confidence interval of − 8.18 to + 8.09 mm. The method is demonstrated in an example data set comparing different interventions for foot drop, and it shows clear differences among no intervention, functional electrical stimulation, and ankle–foot orthosis conditions. This method offers researchers and clinicians a rich understanding of a person’s gait by providing objective 3D foot kinematics and allowing a unique opportunity to view the regions of the foot where minimum clearance occurs. This information can contribute to a more informed recommendation of specific interventions or assistive technology than is currently possible in standard clinical practice.

## Introduction

The measurement of minimum foot clearance during the swing phase of gait is critical in efforts to understand and reduce the risk of trip-and-fall incidents in populations such as older adults, persons with amputation, and persons with gait impairments secondary to neurologic impairment. The concept of minimum foot clearance (mFC) refers to the shortest vertical distance between the foot (including the shoe) and the walking surface during the swing phase of gait. This is a critical event during swing phase because it coincides with the period of maximum forward velocity^1^, making any ground contact a potentially serious trip hazard. High variability in mFC can be a risk factor for scuffing or tripping—events that have been identified as the cause of 53% of falls in older adults^2^. Accordingly, many researchers have focused on developing measurement techniques to estimate foot trajectory and capture mFC accurately^3–6^. The availability of wearable inertial measurement units (IMUs) has improved the portability and cost of gait measurement, allowing real-world data collection.

Researchers have proposed various methods of estimating mFC from wearable sensors; however, such methods are typically limited by the assumption that a given point on the foot, often the toe, is the lowest vertical point relative to the walking surface during swing phase^3–6^. Using a single point underestimates mFC^7^ and studies have found that factors such as paving type, ground slope, walking speed, obstacles, and stairs also affect the location on a person’s foot where mFC occurs^7–10^. Much of the literature examining mFC (or minimum toe clearance) assumes typical swing phase lower extremity kinematics ^3–6^. However, this assumption may not hold in cases of neurologically-rooted gait impairments, which often include excessive inversion and/or lack of controlled dorsiflexion^11,12^. In such scenarios, monitoring only a single point cannot fully capture these multi-planar deviations, potentially leading to inaccurate foot clearance estimates. Further, neurologic swing phase deviations in ankle kinematics are often linked to stance phase stability^13^. Assessment of mFC’s true location can provide clinicians with nuanced insight into gait deviations and the efficacy of related interventions in reducing risks of trips and falls. Methods have been proposed to determine mFC considering more than a single point^7,8,10,14–16^ but these have all been based on in-lab optical motion capture. Current methods, therefore, fall short of an accurate, real-world, representation of mFC and may lead to inaccurate conclusions regarding the biomechanics of gait^17^.

In this work, we propose a method that combines data from a single foot-mounted IMU with a 3D scan of the participant’s shoe/foot to reconstruct and analyze complete shoe movement during the swing-phase of gait. This method can be used to estimate the global x–y–z position of each point on the 3D-scanned shoe, including toe and heel position and any other points of interest. This method treats the participant’s shoe and attached IMU as a rigid body system and is therefore only valid during swing-phase when the foot is unloaded and in the air—making this method suitable for foot clearance estimation.

In addition to describing the method, we validate it against optical motion capture. As it is impractical to view the bottom surface of the foot/shoe using optical motion capture^10^ near ground contact, we instead perform an implicit validation by measuring the height trajectory of a single retroreflective marker. Specifically, we compare the observed height of a toe-marker at key swing-phase moments as captured by an optical motion capture system against the reconstructed toe-marker height trajectory obtained from the IMU + Scan fusion. We report the agreement between measurement techniques with a mixed-effects model for three participants.

Once validated, we demonstrate an application of the method: determining minimum foot clearance in a single participant with foot-drop due to multiple sclerosis (MS) during fatiguing six-minute walk tests (6MWT)^18^. The participant performed three 6MWTs: one with no intervention, one while using a carbon fiber ankle foot orthosis (AFO), and one with a functional electrical stimulation (FES) device. In each 6MWT, we expected mFC to decrease over time as the participant became fatigued, a common symptom of MS. We also hypothesized that the expected decrease in mFC from first to last minute would be greatest in the no-intervention case and less when using both interventions.

## Methods

### Data Collection

**Figure 1:**
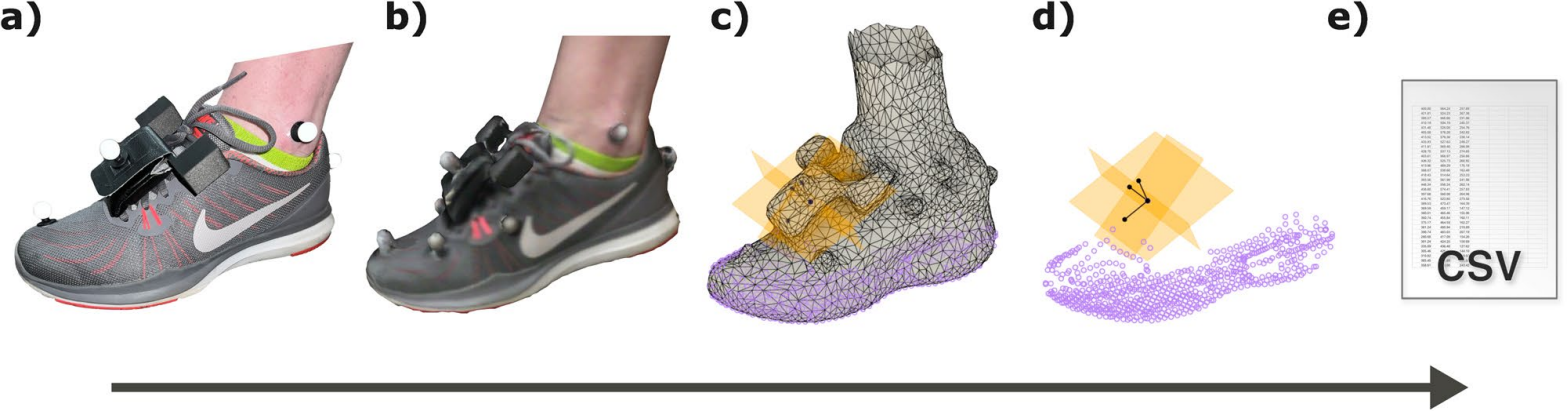
Example of the process to generate the list of coordinates of salient points in the 3D scan’s reference frame. (**a**) Photo of participant’s foot. (**b**) 3D scan of foot. (**c**) Model imported into CAD software with IMU center, axes, and points covering the bottom surface of the shoe. (**d**) Points and orientation vectors (black) shown without model. (**e**) Representation of the CSV file generated from created points.

This study was carried out in accordance with the tenets of the Declaration of Helsinki and with the approval of the University of Wisconsin-Madison Health Sciences Institutional Review Board. Three participants with a diagnosis of foot-drop secondary to Multiple Sclerosis (MS) were included in this pilot study after giving written informed consent according to procedures approved by the University of Wisconsin-Madison Health Sciences Institutional Review Board (HS-2019-0844). We securely placed one IMU sensor (Opal, APDM Wearable Technologies, OR, USA) in a small pouch on each of the participant’s walking shoes and then scanned participant’s feet/shoes with a 3D scanner (Structure Sensor, Occipital Inc., CO, USA) connected to an iPad (Apple, CA, USA) using the companion Occipital "Scanner" app. To facilitate subsequent identification of the position and orientation of the IMU during the scan, we placed a 3D-printed fixture inside the pouch tightly fitted to the IMU, with locating "ears” protruding outside the pouch, see Fig. 1. This fixture was removed for the data collection to avoid disrupting the participants’ gait.

We placed reflective markers on the participants according to a modified Helen Hayes marker set. In this data validation, only information from the toe marker was considered. The toe marker was placed on top of the shoe near the furthest anterior point of the toe, along the mediolateral midline of the shoe.

The participants walked back and forth in the motion capture lab while body kinematics were recorded using optical motion capture (twelve Optitrack Prime 13 cameras, Natural-Point, Inc., Corvallis, OR, USA). Motion capture and IMU data were recorded at 200 and 128 Hz, respectively.

### Foot IMU movement reconstruction

The trajectory of the foot IMU was reconstructed using IMU-based Pedestrian Dead Reckoning (PDR), which computes position and orientation from raw acceleration and gyroscope data using an enhanced integration-based reconstruction method. The reconstruction method determines the timing of stance-phase zero-velocity updates (ZUPT) by thresholding the norm of the accelerometer signal and the gyroscope signal, similar to^3,19–23^, then smoothing the ZUPT index by deleting ZUPT periods that were shorter than 15 samples to remove any potential erroneous stance phases. In addition to the traditional IMU motion reconstruction method used in^3,19–23^, we improved the reconstruction accuracy on height by estimating acceleration bias and the impulsive velocity error at heel strike on each stride, and removing the error caused by these two factors. Further details on the reconstruction can be found in our work by Wang et al.^24^. The following data obtained from the reconstruction were used in the analysis: global X–Y–Z coordinates of the IMU, rotation matrix indicating the global orientation of the IMU, and indices representing the ZUPT periods.

### 3D scan processing

Using CAD software (Fusion 360, Autodesk), we imported scans of the participant’s feet (shoes, IMU, and the 3-D printed fixture were worn during the scan, Fig. 1b) and defined landmarks at the IMU’s center and axes. These salient points were located based on the fixture’s rectangular “ears” (visible in Fig. 1c) that were used to establish datum planes and edges. Care was taken to ensure that the datum planes were perpendicular to each other. The locations of the toe and heel were determined using several steps. Initially, a line was drawn to connect the medial edges of the toe and heel, as viewed from the transverse plane. Subsequently, two planes were identified perpendicular to that line at the farthest anterior and posterior extremes of the foot. The toe and heel points where then placed on these extreme points.

**Figure 2:**
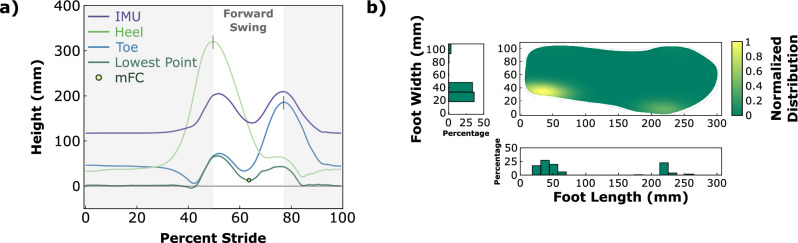
(**a**) Height of different important points during a representative stride as constructed by the proposed method. Strides are segmented by ZUPT periods (e.g. 0% is the start of the ZUPT period). (**b**) location of the lowest point on the foot during late-swing (last third of forward swing)—seen from above in the main plot, with histograms representing frequency along foot’s x- and y-axes.

Once the toe and heel points were marked, we projected a grid of points that covered the bottom surface of the shoe to generate a point cloud representation of the foot’s geometry relative to the IMU’s coordinate system. Figure 2a illustrates the heights of the toe, heel, and IMU center point for a single stride. For validation against motion capture, an additional point was placed at the center of the reflective toe marker, which was also visible in the scan. Once these points were created in the CAD software, they were exported via a custom Fusion 360 script (Python) to a comma-separated values (CSV) file. Figure 1 shows a graphical representation of this process.

### Reconstructing whole foot movement in the world frame

To determine the location of the foot’s points in the world frame, we combined the global (‘world’ frame) pose of the IMU from the reconstruction with the positions of salient points on the foot in the 3D scan’s reference frame (‘scan’ frame). This calculation is expressed in Eq. 1, where the nomenclature is as follows: the left superscript indicates the current frame in which a vector is expressed, or the resulting frame after a matrix transformation; the left subscript indicates the frame in which vectors are expressed before that matrix transformation; and any right-side subscripts serve as descriptors.

1 $$\begin{array}{*{20}c}    {^ {\text{world}}  }  \\      \\      \\   \end{array}\!\!\! \left[ {\begin{array}{*{20}c}    {x_{i} }  \\    {y_{i} }  \\    {z_{i} }  \\   \end{array} } \right]  = \begin{array}{*{20}c}    {_ {\text{world}}  }  \\    {^ {\text{IMU}}  }  \\   \end{array}\!\! R(k)~*\begin{array}{*{20}c}    {_ {\text{IMU}}  }  \\    {^{\text{scan}} }  \\   \end{array}\!\! R*\begin{array}{*{20}c}    {^ {\text{scan}}  }  \\      \\      \\   \end{array}\!\!\! \left[ {\begin{array}{*{20}c}    {x_{i} }  \\    {y_{i} }  \\    {z_{i} }  \\   \end{array} } \right]$$

The rotation matrix $_{{{\text{scan}}}}^{{{\text{IMU}}}} R$ transforms points from the scan frame to the IMU’s frame, utilizing the orientation vectors obtained from the fixture in the scan (Fig. 1D). The matrix $$ \begin{array}{*{20}c}    {_{{{\text{world}}}} }  \\    {^{{{\text{IMU}}}} }  \\   \end{array}\!\! R    $$ is extracted from the IMU reconstruction at sample *k*, representing the orientation of the IMU in the world frame and used to transform vectors from the IMU frame to the world frame.

When observing the outcome of this procedure we noted that the foot appeared to be slightly tilted, particularly at times when it was anticipated to be flat, such as during stance phase. To correct for this erroneous tilt, we introduced a correction as detailed below. Omitting this correction resulted in a foot tilt error up to ± 6° relative to floor during the ZUPT period. This error may stem from slight errors in processing the scan in the CAD software or from the IMU shifting slightly in the pouch after removing the fixture. The correction involved finding the rotation matrix, $$ \begin{array}{*{20}c}    {_{{{\text{world}}}} }  \\    {^{{{\text{world}}}} }  \\   \end{array}\!\! R_{{flat}} $$, that makes the foot parallel to the global x–y (floor) plane when it is on the floor (see Eq. 2). These calculations were performed at the first instant of the first ZUPT period of the bout in question, ‘*ZUPT*_1_’. To estimate$$ \begin{array}{*{20}c}    {_{{{\text{world}}}} }  \\    {^{{{\text{world}}}} }  \\   \end{array}\!\! R_{{flat}} $$, we used an optimization procedure to find a plane flush against the bottom of the point cloud, then computed the minimal rotation that would cause that plane (and therefore the foot) to rest flat on the floor. Further details on this optimization procedure can be found in Supplement A.

2 $$ \begin{array}{*{20}c}    {^ {\text{world}}  }  \\      \\      \\   \end{array}\!\!\! \left[ {\begin{array}{*{20}c}    {x_{{ZUPT_{1} }} }  \\    {y_{{ZUPT_{1} }} }  \\    {z_{{ZUPT_{1} }} }  \\   \end{array} } \right]_{{flat}}  = ~\begin{array}{*{20}c}    {_ {\text{world}}  }  \\    {^ {\text{world}}  }  \\   \end{array}\!\! R_{{flat}} \begin{array}{*{20}c}    {^ {\text{world}}  }  \\      \\      \\   \end{array}\!\!\! \left[ {\begin{array}{*{20}c}    {x_{{ZUPT_{1} }} }  \\    {y_{{ZUPT_{1} }} }  \\    {z_{{ZUPT_{1} }} }  \\   \end{array} } \right]   $$

Combining Eqs. 1 and 2, we have the equation that transforms the points in the scan frame to the world frame, corrected (Eq. 3), where *k*_*ZUPT*__1_ is the first instant of the first ZUPT period in the bout.

3 $$ \begin{array}{*{20}c}    {^ {\text{world}}  }  \\      \\      \\   \end{array}\!\!\! \left[ {\begin{array}{*{20}c}    {x_{{ZUPT_{1} }} }  \\    {y_{{ZUPT_{1} }} }  \\    {z_{{ZUPT_{1} }} }  \\   \end{array} } \right]_{{flat}}  = \begin{array}{*{20}c}    {_ {\text{world}}  }  \\    {^ {\text{world}}  }  \\   \end{array}\!\! ~R_{{flat}} ~*\begin{array}{*{20}c}    {_ {\text{world}}  }  \\    {^ {\text{IMU}}  }  \\   \end{array}\!\! R(k_{{ZUPT_{1} }})~*\begin{array}{*{20}c}    {_ {\text{IMU}}  }  \\    {^ {\text{scan}}  }  \\   \end{array}\!\! ~R*~\begin{array}{*{20}c}\!    {^ {\text{scan}}  }  \\      \\      \\   \end{array}\!\!\! \left[ {\begin{array}{*{20}c}    {x_{{ZUPT_{1} }} }  \\    {y_{{ZUPT_{1} }} }  \\    {z_{{ZUPT_{1} }} }  \\   \end{array} } \right]$$

To streamline the process and avoid recalculating $$    \begin{array}{*{20}c}    {_{{{\text{world}}}} }  \\    {^{{{\text{world}}}} }  \\   \end{array}\!\! R_{{flat}} $$ at each instant, we introduced ${R}^{*}$ in the local IMU frame at ZUPT_1_ and applied it to all samples. In this case, the $$ \begin{array}{*{20}c}    {_{{{\text{world}}}} }  \\    {^{{{\text{IMU}}}} }  \\   \end{array}\!\! R(k_{{ZUPT_{1} }}) $$ is the rotation from the IMU reconstruction at the first instant of the first ZUPT period. The resulting equation used is as in Eq. 4.

4 $$\begin{array}{*{20}c}    {^ {\text{world}}  }  \\      \\      \\   \end{array}\!\!\! \left[ {\begin{array}{*{20}c}    {x_{i} }  \\    {y_{i} }  \\    {z_{i} }  \\   \end{array} } \right]_{{flat}}  = ~\begin{array}{*{20}c}    {_ {\text{world}}  }  \\    {^ {\text{IMU}}  }  \\   \end{array} \!\!R(k)~\,*\,~\left( {R^{*} \,*\,~\begin{array}{*{20}c}    {_ {\text{IMU}}  }  \\    {^ {\text{scan}}  }  \\   \end{array}\!\! R} \right)\begin{array}{*{20}c}    {^ {\text{scan}}  }  \\      \\      \\   \end{array}\!\!\! \left[ {\begin{array}{*{20}c}    {x_{i} }  \\    {y_{i} }  \\    {z_{i} }  \\   \end{array} } \right] $$

${R}^{*}$ Is derived by comparing the matrices in Eqs. 3 and 4 at sample time *k*_*ZUPT*__1_:

5 $$ \begin{array}{*{20}c}    {_{{{\text{world}}}} }  \\    {^{{{\text{IMU}}}} }  \\   \end{array}\!\! R(k_{{ZUPT_{1} }})\,*\,R^{*} ~ = ~\begin{array}{*{20}c}    {_{{{\text{world}}}} }  \\    {^{{{\text{world}}}} }  \\   \end{array}\! R_{{{\text{flat}}}} ~\,*\,~\begin{array}{*{20}c}    {_{{{\text{world}}}} }  \\    {^{{{\text{IMU}}}} }  \\   \end{array}\!\! R(k_{{ZUPT_{1} }}) $$

6 $$ \therefore R^{*} ~ = ~\left( {\begin{array}{*{20}c}    {_{{{\text{world}}}} }  \\    {^{{{\text{IMU}}}} }  \\   \end{array}\!\! R(k_{{ZUPT_{1} }})} \right)^{T} ~\,*\,~\begin{array}{*{20}c}    {_{{{\text{world}}}} }  \\    {^{{{\text{world}}}} }  \\   \end{array}\! R_{{flat}} ~\,*\,\begin{array}{*{20}c}    {_{{{\text{world}}}} }  \\    {^{{{\text{IMU}}}} }  \\   \end{array}\!\! R(k_{{ZUPT_{1} }})   $$

7 $$ \begin{array}{*{20}c}    {_{{{\text{IMU}}}} }  \\    {^{{{\text{IMU}}}} }  \\   \end{array}\!\! R_{{{\text{flat}}}} ~ = ~R^{*}  $$

### Determining floor height

When the IMU trajectory is reconstructed, the absolute zero height is initialized as the global z position of the IMU at the beginning of the trial and during subsequent ZUPT periods. This definition results in the foot appearing to penetrate the floor throughout stance phase. To more correctly estimate the position of the floor, we shifted all points upward by a vertical offset calculated for each trial. We calculated this vertical offset by measuring the height of the lowest point on the foot at every sample during each ZUPT period. For each stride, we calculated the median of these points during ZUPT. Finally, we averaged these median heights to obtain the vertical offset specific to that trial. We then applied this trial-specific vertical offset to all points on the foot, including the IMU’s position. This adjustment ensured that our data more accurately approximated the true height of the floor.

### Calculating minimum foot clearance

Once the trajectory of the whole foot is reconstructed, we determine the instantaneous foot clearance as the height of the lowest point (LP) at any instant (see the ‘Lowest Point’ trajectory in Fig. 2a). We further determine the overall minimum foot clearance (mFC) by identifying the lowest value of foot clearance during forward swing, defined as the period from maximum heel height to maximum toe height during swing phase (the non-shaded region of Fig. 2a). These points represent the extremes of foot movement along its swing path and approximate the period of swing where the foot has an anterior velocity relative to the person’s orientation in the world. Note that this definition of forward swing may not hold for all gait patterns, particularly in cases of impairment.

The formal mathematical definition of this process is as follows. For each stride, let $K$ be the total number of data samples within that stride, and $N$ be the total number of points in the 3D scan point cloud. The position vector of a point with index $i$ within the point cloud at sample $k$ is stored as $pointCloud(i,k)$; the height of that point is $pointClou{d}_{z}\left(i,k\right)$; the index that refers to the heel point is ${i}_{Heel}$; the index that refers to the toe point is ${i}_{Toe}$. Processing to determine mFC uses the following steps:

8 $$LP\left(k\right)=\underset{1\le i\le N}{\mathit{min}}pointClou{d}_{z}\left(i,k\right)$$

9 $${k}_{maxHeel}=\underset{1\le k\le K}{\mathit{argmax}}\,pointClou{d}_{z}\left({i}_{Heel},k\right)$$

10 $${k}_{maxToe}=\underset{1\le k\le K}{\mathit{argmax}}\,pointClou{d}_{z}\left({i}_{Toe},k\right)$$

11 $$mFC = \underset{{k}_{\mathit{maxHeel}}\le k\le {k}_{\mathit{maxToe}}}{\mathit{min}}LP(k),$$

Where ${k}_{maxHeel}\le k\le {k}_{maxToe}$ represents the period of forward swing described above.

### Late-swing minimum clearance region

During the final third portion of forward swing (“late-swing”) for each analyzed gait cycle, the lowest point on the foot relative to the ground was determined. In this way, efficacy of foot-drop interventions for improvements in sagittal plane ankle kinematics could be compared for the portion of swing where the normal kinematics would predict the greatest dorsiflexion to occur^25^. To visualize the density and distribution of the foot’s lowest points during this period of the gait cycle during a bout of walking, we constructed a density histogram of where these lowest points occurred on the foot. We used a Gaussian kernel centered at the lowest point from each sample to smooth nearby points into interpretable regions. First, a mesh grid was set up over the surface of the sole of the shoe, and at each sample of the trajectory a single point was determined as the lowest point on the foot. The values for the final third of forward swing were isolated. Next, Gaussian values were iteratively added around the lowest grid points based on this trajectory, generating a density plot across the whole grid of the foot sole. Finally, each grid point’s value was normalized by dividing by the maximum value existing on the grid. This density plot is viewed from above as a heat map (see Fig. 2b); one-dimensional histograms were also generated to view the distribution of lowest points along the foot’s x- and y- axes.

### Validation Method

To gain confidence in the height estimated for points in the scan using this method, we validated the reconstruction against the “gold standard,” motion capture (Mocap). To do so, we validated the height of the toe marker as measured by Mocap and estimated by the IMU + Scan method. This is an indirect validation approach for mFC, used because measuring the ‘lowest point’ of the shoe at each instant with motion capture is a very involved process^10^.

**Figure 3:**
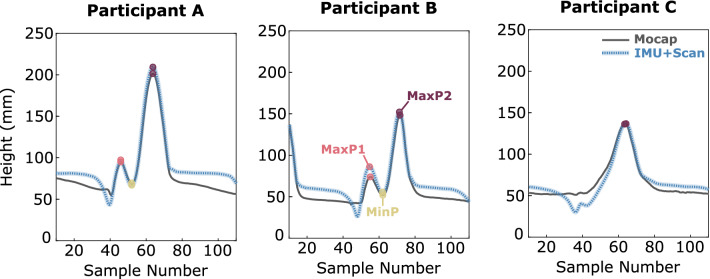
Representative toe marker trajectories and key points for participants A, B, and C.

As previously mentioned, we exported the location of the center of the reflective toe marker from the 3D scan. The trajectory of this point was transformed into the world frame alongside the rest of the point cloud. Concurrently, we extracted the trajectory of the toe marker from the Mocap data during the in-lab walks. This Mocap trajectory data was downsampled via interpolation to match the IMU sampling rate of 128 Hz. We matched the strides from the IMU reconstruction to the Mocap data and compared the height of key maxima and minima of toe marker height during swing phases. Toe marker trajectories from Participants A and B followed M-shaped paths during swing while Participant C had a single maximum only. Because of this, for participants A and B each swing phase contained three key points: the two maxima (MaxP1 and MaxP2) and the minimum between them (MinP). Participant C’s swing phase contained a single key point at the maximum (MaxP2). The measurement of these key points will be referred to as “IMU + Scan Height” and “Mocap Height”. Figure 3 shows representative toe marker trajectories and key points for participants A, B, and C as determined by the proposed IMU + Scan reconstruction and by motion capture. Note that the sample number axis indicating timing in this figure (Fig. 3) is arbitrary because the measures of interest are determined by height, not time.

### Statistics

To determine agreement between our measurements, we used a mixed-methods limits of agreement approach^26^. We first calculated the height of the toe marker as measured by both the motion capture system, ‘Mocap’, and our proposed IMU method, ‘IMU + Scan’. Each measurement was grouped by participant (participantID) and by stride (strideID). For example, in Fig. 3, one stride is shown—therefore, the six highlighted points, (MaxP1, MinP1, and MaxP2 for the IMU + Scan and Mocap) would share the same strideID. We then calculated the difference between each sample of IMU versus Mocap height. Finally, we fit a linear mixed-effects model to this difference, *IMUScan_Height* –*Mocap_Height*, with random effects of strideID nested below participantID. The ‘lme’ function from the ‘nlme’ package^27^ in R software^28^ was used as in Eq. 12:

12 $${\text{lme}}(\left( {{\text{IMUScan}}\_{\text{Height}}{-}{\text{Mocap}}\_{\text{Height}}} \right)\,\sim \,{1},{\text{ random}}\, = \,\,\sim \,{1}|{\text{participantID}}/{\text{strideID}})$$

With this model we found the bias (e.g. intercept) and the limits of agreement, i.e. the 95% confidence interval of the bias.

## Validation Results

**Figure 4:**
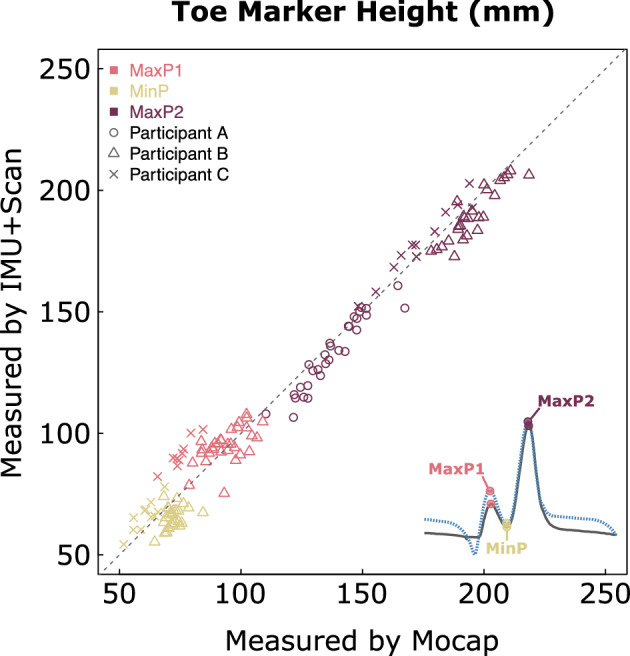
Absolute agreement chart showing the difference in toe marker height measurements, Mocap – IMU + Scan. Dashed line represents ideal agreement (y = x).

The results of the mixed-effects limits of agreement analysis (Table 1) revealed a bias of − 0.05 mm with a 95% confidence interval (CI) of − 8.18 to + 8.09 mm. That is, on average the IMU + Scan measured the toe marker height as 0.05 mm lower than the motion capture system and and we can be 95% confident that the true bias of the IMU + Scan is up to 8.09 mm above or 8.18 mm below the motion capture system, when accounting for the random effects of stride and participant. Figure 4, shows all measurements considered in the analysis as determined by the motion capture system and proposed IMU method.

**Table 1:** Results of the mixed-effects limits of agreement analysis, measurements are in millimeters.

	Value
Bias	− 0.05
CI lower limit	− 8.18
CI upper limit	8.09
R^2^	0.983
Sample size	146
Alpha	0.05

## Example Use Case

A benefit to this method is the ability to determine minimum foot clearance (mFC) outside of the lab with minimal sensors. In this example, mFC was defined as the lowest height of any point on the foot during the forward swing, defined between the maxima of heel and toe height during swing phase. This method is especially relevant to studying impaired walking, in which the minimum foot clearance may be different from the minimum toe clearance (MTC) calculated using other methods. For example, the minimum foot clearance may occur at the lateral or medial edge of the foot. The participants in this study were persons with foot-drop due to multiple sclerosis–a population for whom this method is particularly applicable.

**Figure 5:**
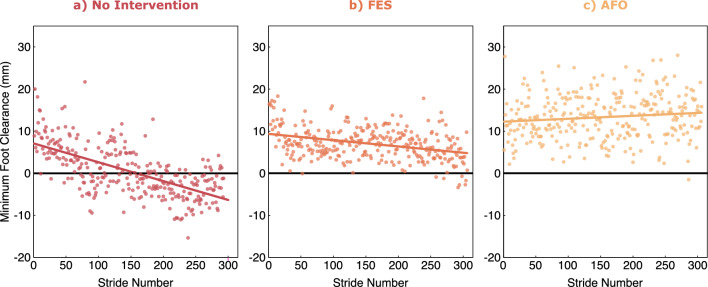
Minimum foot clearance for participant A during a 6MWT under three conditions: (**a**) no intervention (**b**) using an FES and (**c**) using an AFO.

To demonstrate the method, we evaluated the mFC of Participant A throughout three fatiguing six-minute walk tests (6MWT^18^) performed in different conditions on different days. The first 6MWT was performed with no intervention. The second and third 6MWTs were performed with two interventions commonly prescribed for MS-related foot-drop: a functional electrical stimulation (FES) device (L300 Go, Bioness, CA, USA) and a flexible carbon fiber ankle–foot orthosis (AFO) (Sprystep, Thuasne, CA, USA). These two interventions were each tested after an acclimation period of at least five days of at-home use. Figure 5 shows minimum foot clearance under each of the three conditions. The 6MWT is designed to cause fatigue, especially in participants with conditions like MS^29^. Therefore, it is unsurprising that the whole foot minimum clearance decreased significantly (p < 0.001) over time in the no-intervention case. In this case, the participant’s foot clearance also decreased significantly when using the FES device (p < 0.001). However, when using the AFO, there was a borderline significant increase in clearance (p = 0.046).

**Figure 6:**
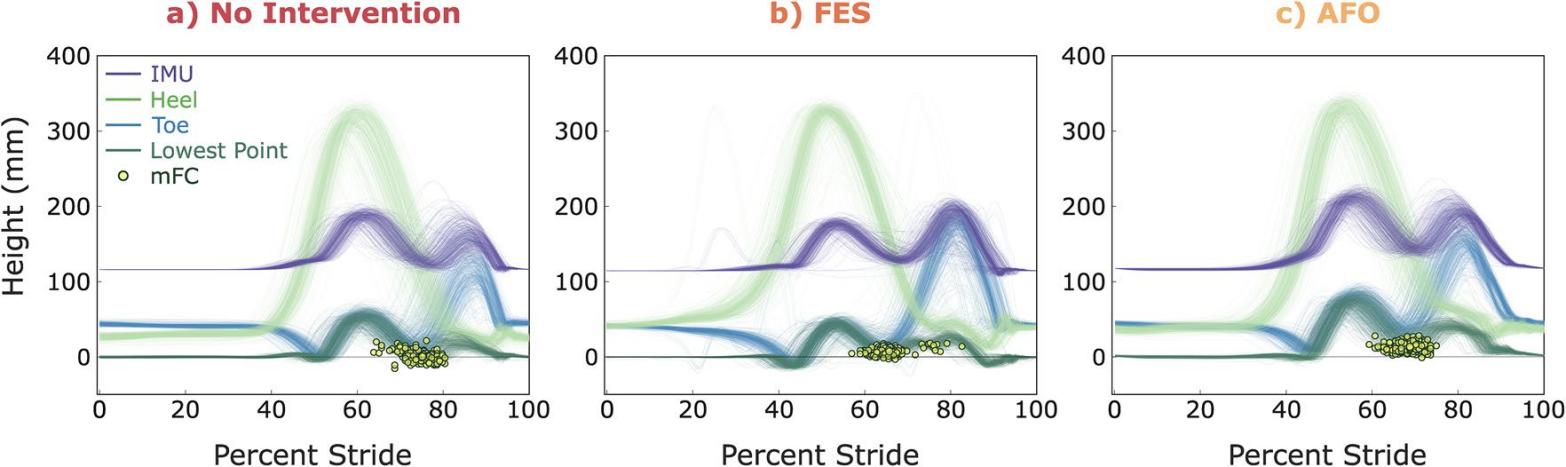
Heights of different points on the foot for participant A during a 6MWT under three conditions: (**a**) no intervention (**b**) using an FES and (**c**) using an AFO. Each thin trace represents a single stride; strides are segmented by ZUPT periods (e.g. 0% is the start of the ZUPT period) and normalized to percent stride. For reference toe-off is roughly 45% and heel-strike is roughly 85%.

To understand the results further, Fig. 6 shows the traces of the height of the IMU, heel, toe, and lowest point during the 6MWT for each condition. A video animating the reconstruction of 5 steps in each condition is provided as a supplemental video (Supplement B). Each thin trace represents a single stride. In addition, mFC is marked on the lowest point curve to show the timing of mFC within each stride. Comparing the different conditions, it is evident that the No Intervention case shows a more varied and lower toe clearance than the others, and mFC occurring later during forward swing. Within this variability, mFCs that occur later also have lower clearance than those that occur earlier. In contrast, when using the FES device, minimum toe clearance appears to be increased and mFC timing is more spread out. The lowest point curve looks bumpier, which may reflect the effects of the electrical stimulation. Finally, the AFO shows a less variable and greater mFC.

**Figure 7:**
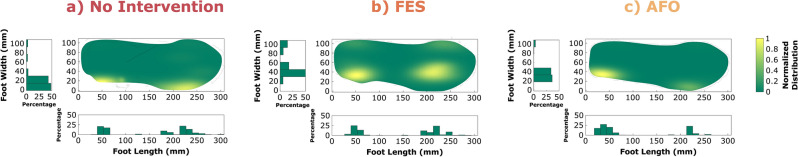
Gaussian heatmaps with histograms, each representing location on foot of lowest point during late-swing (final third of forward swing) in final minute of 6MWT. Three conditions represented: (**a**) No Intervention (**b**) FES and (**c**) AFO. Key provided for density heatmap at far right of figure.

In addition to calculating forward swing mFC, we observed the locations on the foot where late-swing mFC occurred. Figure 7 displays the probability distribution of mFC occurrence for each of three experimental conditions (No Intervention, FES, and AFO). Clear variations in mFC distribution are evident when comparing between interventions. These variations support the expectation that our methods may be useful in analyzing differences in mFC as they relate to gait impairment and intervention. To understand how foot-drop interventions influence mFC, we restricted data on Fig. 7 to the latter third of the forward swing period where typical kinematics would predict that plantarflexion would not be the predominant ankle position^30^.

## Discussion

We proposed a method to determine minimum foot clearance by pairing data from a single IMU with a personalized 3D foot scan. As this is a lightweight and wireless approach, data can easily be collected outside of the lab. Furthermore, this method measures *whole-foot* mFC, acknowledging that not all movement in gait is sagittal and that it takes more than heel and toe height to describe foot clearance. Reconstruction of free-living, full foot trajectory, could allow researchers and clinicians to find and study important or unusual events, such as foot scuffing and natural trips and falls rather than those induced in the laboratory (see Supplement B for animations of the three 6MWT conditions and Supplement C for an example of a stumble identified via the proposed whole foot reconstruction). As this method uses fully portable data collection methods, it is possible to reach strong conclusions in variable data sets due to large stride count.

This method may also be used to reconstruct and analyze foot clearance throughout the entire swing phase, rather than simply recording a single local minimum, mFC. This is important in gait cycles with no MTC events, as that of Participant C (Fig. 3), or in cases with multiple minima. Studies have found that gait cycles without MTC events are more prevalent in older participants (2.9% of all gait cycles in young participants; 18.7% in older participants)^31^ and when walking over surfaces with obstacles (2% of all gait cycles with no obstacles; 20% with obstacles)^10^. Work by Byju et al.,^16^ showed that using the trajectory of the instantaneous anterior-most virtual marker on the bottom edge of the foot across the entire swing phase resulted in a more accurate prediction of tripping compared to using the trajectory of a single physical marker on top of the toes. The proposed method allows for such analysis during long over-ground walks, potentially in clinical spaces, by using a single foot mounted IMU rather than a traditional motion capture system.

Our overall results are promising, showing a very small bias, less than 0.05 mm, with 95% confidence interval of − 8.18 to + 8.09 mm. This level of uncertainty may be significant, as previous studies have shown that typical minimum clearance ranges from 11 to 15 mm^32^. Nonetheless, the example data show that this method is enough to measure changes over time and compare among interventions. The most similar prior work, by Mariani et al.^17^ determined heel and toe clearance with a single IMU sensor. Their approach estimated the heel and toe based on a kinematic model that used an algorithm to detect toe-off and heel-strike events. They found a mean difference ± standard deviation of 41 ± 23 mm for maximal heel clearance and 13 ± 9 mm for minimal toe clearance (MinP) compared to motion capture. The method proposed in this paper shows a reduced mean difference, 0.05 mm, and a reduced uncertainty, approximately ± 8 mm CI. Furthermore, it is not reliant on the ability to detect toe-off and heel-strike events, which can prove challenging, especially in impaired populations.

It is important to note that the full foot trajectory is only valid during swing phase—when the foot is unloaded and off the ground. This is because the 3D scan enforces an assumption that the foot is a rigid body. Due to this, the method does not replicate the foot bending at the toe joint during pre-swing, resulting in a phenomenon where the toe height sharply decreases (and typically appears to penetrate the ground) near the end of stance phase (see the Toe trajectory in Fig. 6). Discretion must be used in interpreting findings derived via this method, particularly in situations where the foot may deform during swing phase unexpectedly, e.g. encountering obstacles, stumbles, falls, and gait impairments. To capture the bend at the toe joint, it may be possible to utilize additional IMUs and separate the scan into sections, as was done with motion capture data in^10^.

In the example use case, the proposed method was used to compare different interventions for foot drop in a fatiguing 6MWT. For this participant, the AFO condition showed constant or slightly increasing mFC over time, whereas in the No Intervention and FES conditions, mFC decreased over time (Fig. 5). mFC decreased most severely in the No Intervention condition, as expected. In the analysis of the late-swing mFC location (Fig. 7), the No Intervention condition exhibited the highest total percentage of mFC occurrence in the forefoot region compared to the other two conditions. In contrast, the AFO condition primarily showed mFC occurrences in the hindfoot region, indicating better control of foot drop. The FES condition was between these two. Among the three conditions, the FES condition demonstrated the most variability in mFC location along the medial–lateral axis. This observation aligns with the expectation of greater control of frontal plane kinematics provided by a rigid AFO compared to an FES device. Ideally, the FES device should induce dorsiflexion without causing frontal plane motion. Nevertheless, given the imprecision of surface electrodes, mFC occurrences at the medial border of the foot might arise due to co-stimulation of peroneus tertius or other ankle evertors. In a clinical context for this single subject, such findings would motivate re-assessment of FES fit and stimulation parameters to minimize unintended frontal plane motion and reduce the risk of initial contact in an ankle position that may lead to instability or injury. Findings such as this demonstrate how knowledge of mFC location can provide valuable insight for clinical decision making in selection of and adjustment to foot drop interventions.

**Figure 8:**
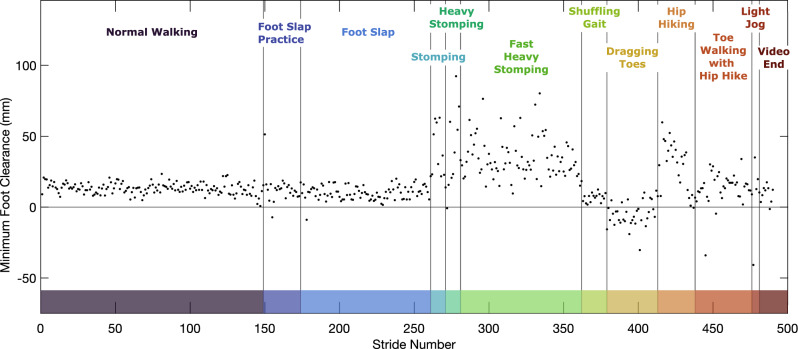
Minimum foot clearance (right foot) when imitating different walking styles.

During the majority of the no intervention 6MWT, and in select strides in the FES and AFO conditions, mFC was negative. In initial tests, negative clearance was observed as an artifact of the person’s foot coming into contact with the floor during swing phase, for example during a trip or stumble (See Supplement C). The reasons for negative clearance may be two-fold—during this mid-swing contact the shoe deforms, which is not captured by our method that assumes a rigid body, and this additional impact causes a spike in acceleration potentially introducing error into the reconstruction^24^. When observing findings from our MS cohort, negative clearance was more prevalent, especially as participants grew more fatigued. To understand if negative clearance was indicative of a certain gait style, we collected a sample imitating different walking styles, shown in Fig. 8. This demonstration is discussed in more detail in Supplement D, including videos. Walking with dragging toes, particularly when the toe would bounce on the floor during swing, resulted in findings of negative clearance similar to those in the example use case.

The ability to determine what points on the foot are most at risk of scuffing or tripping could lead to new interventions or a better understanding of how to choose among several existing interventions. Current interventions for foot-drop utilizing functional electrical stimulation, such as the L300 Go (Bioness, CA, USA) and WalkAide (Accelerated Care Plus, NV, USA), could be improved by user-specific information about foot positioning through forward swing paired with population-level information regarding the kinematics of specific foot regions and how they present as the lowest region in both typical and atypical gait.

The utility of this method is significantly enhanced by the incorporation of user-specific 3D scans. This user-centered adaptability positions our approach to be applicable to a wide range of populations and with a variety of orthopedic devices having arbitrary geometry. Notably, the ability to reconstruct foot movement despite nonstandard foot geometry could be valuable in the cases of persons with amputation, limb deficiency or dysmelia, ankle arthrodesis, acquired foot deformities, peripheral neuropathy impacting ankle control, stroke, cerebral palsy, muscular dystrophy, and other conditions which impact typical gait.

We posit that this approach has merit as a unique way to measure mFC, and to do so outside of the lab. Furthermore, this method offers researchers and clinicians a rich understanding of a person’s gait by providing objective 3D foot kinematics that can be measured over long or short periods of time. In current clinical practice, objective measurement is limited mainly to short periods of walking in clinic. Many neurologic conditions which impact gait are accompanied by increased fatiguability, resulting in changes in gait kinetics over periods of walking that may not be clinically feasible due to space and time limitations. This method also allows a unique opportunity to view the regions of the foot where minimum clearance occurs, which can provide insight to clinicians in the degree of ankle control currently achieved vs. that desired, in all planes. This information can contribute to a more informed recommendation of specific interventions or assistive technology than is currently possible in current standard clinical practice.

## Limitations and Potential Improvements

With the development of this proposed method, it is essential to acknowledge its boundaries. As previously mentioned, this method incorrectly assumes that the foot is a rigid body. This results in inaccurate representations during stance phase and any other time where the foot makes contact with the floor. This also limits the applicability of the method to bare feet, as the foot may flex even while unloaded without a semi-rigid shoe surrounding it. Furthermore, the scan of the shoe is taken with the 3-D printed fixture within the shoe pouch which is later removed. This may cause the IMU to have moved within the pouch during the data collection resulting in a slightly inaccurate estimation of the IMU center and orientation. The effects of such inaccuracy would be greater for IMU orientation than position; this is why we allowed a minor orientation adjustment to ensure a level shoe during stance phase. Nevertheless, slight misalignment may remain, and future techniques to eliminate it would be a valuable improvement.

Another consequent issue of the rigid body assumption arises in the definition of the floor’s height. In the proposed method the floor’s vertical position relative to the foot was established during the ZUPT period, when shoe is deformed by body weight, resulting in compression of the sole. This may cause our estimate of the floor to be lower than the true floor by an unknown amount that is dependent on deformations of the foot and shoe. An alternative approach that we tested was setting the floor as the lowest part on the back third of the shoe at the very end of ZUPT when we assume that the heel is just lifting off the ground and the heel would be unloaded. However, at the end of ZUPT and the few subsequent frames there is a rapid change in z-height. Because of this we determined that it was not a repeatable, reliable approach. To improve upon the proposed method, it may be possible to experimentally capture the change in IMU height above the floor due to loading and adjust the floor height accordingly.

When reporting the accuracy of our validation it is important to note that the amount of error is dependent on many factors including the accuracy of the IMU trajectory reconstruction, the quality of the 3D scan and defined points, and the degree to which the IMU is rigidly attached to the shoe in question. To obtain best results it is important to take care when preparing to scan—ensure that the scanner is calibrated and that the scanning fixture is large enough to be clearly observed during processing. The pouch holding the IMU should be attached securely for the duration of the data collection. Alternatively, using a built-in IMU could eliminate uncertainty in IMU position in the scan and keep it firmly in place reducing variability.

The validation between the motion capture and the IMU-scan proposed method was conducted using only the vertical position of the reflective marker on the participant’s shoe. A major application of the proposed method is to understand *whole-foot* ground clearance, which is not directly measured in our motion capture collection. Indirect validation was chosen because it would be very difficult to arrange optical motion capture of all shoe-bottom points throughout the movement; to do so would require camera views at and below the ground. Nonetheless, the location of the reflective marker was determined using the same process as any other point on the shoe and should therefore offer similar confidence.

## Conclusion

In this paper, we proposed a method to reconstruct the swing-phase trajectory of all the points on the surface of a person’s foot from a single, foot-mounted IMU and a 3D-scan of the foot. This can be used to estimate not only toe and heel clearance but also the instantaneous mFC—e.g. the height of the lowest point on the foot at each instant. The validation showed that our proposed method is highly correlated to measurements by motion capture. The limits of agreement showed that the method may over- or under-estimate the height of the foot by up to 8.1 mm. The measurement is sensitive to different interventions and to changes in mFC over time. Negative estimated mFC appears to indicate contact of the foot with the floor, which may be of specific interest in clinical cases. The use of relatively simple and deployable tools (IMU, 3D scanner) and the prospect of automated analysis may make this method useful in clinical gait analysis and prescription practices.

## Supplementary Information

Supplementary Information.

Supplementary Video 1.

Supplementary Video 2.

Supplementary Video 3.

## Data Availability

The datasets and source code used and/or analyzed in this work and its supplementary information files are available from the corresponding author on request.
