# Supplementary material for: Estimation of minimum foot clearance using a single foot-mounted inertial sensor and personalized foot geometry scan
Source: Sci Rep. 2024 Jun 13;14:13640. doi: 10.1038/s41598-024-63124-6 (PMC11637083; doi:10.1038/s41598-024-63124-6)
Supplement: Supplementary file 1 — Supplementary Information. [file 41598_2024_63124_MOESM1_ESM.pdf]

Supplemental Information: Estimation of minimum foot clearance using a single foot-mounted inertial sensor and personalized foot geometry scan

Katherine Heidi Fehr*[1], Jennifer Nicole Bartloff[2], Yisen Wang[2], Scott Hetzel[3], and Peter G. Adamczyk[2]

*[1] kfehr@wisc.edu, Mechanical Engineering Department, University of Wisconsin–Madison, Madison, WI
[2]Mechanical Engineering Department, University of Wisconsin–Madison, Madison, WI
[3]Department of Biostatistics and Medical Informatics, University of Wisconsin-Madison, Madison, WI

Supplement A: Optimization procedure to determine ${}^{world}_{world}R_{flat}$

The following procedure was used to estimate ${}^{\mathrm{world}}_{\mathrm{world}}R_{flat}$, the rotation of the reconstructed foot point cloud that brings the plane of the shoe sole into contact with level ground. To make an initial guess at the shoe sole plane, we used a loss function to minimize the distance from a plane to the point cloud, while strongly penalizing points beneath the plane. However, the initial guess could sometimes result in an inverted or everted plane if the medial or lateral side of the foot had more points. To address this issue, we removed points more than 2 cm above the initial guess and divided the point cloud into quadrants, starting with the point farthest from the centroid, typically either the toe or heel. To shorten compute time, we randomly removed points in each quadrant until 40 points per quadrant remained. Finally, we determined all planes possible from combinations of three points from the reduced, 40-point-per-quadrant cloud and chose the plane that minimized the original loss function.

Using this plane, we calculated the rotation matrix, ${}^{\mathrm{world}}_{\mathrm{world}}R_{flat}$ that corrects the foot's point cloud from its initial orientation to an orientation in which the plane is parallel to the global x-y (floor) plane. This correction was constrained using a Rodrigues angle definition to be orthogonal to the yaw/heading direction and affect only pitch (dorsiflexion/plantarflexion) and roll (inversion/eversion) angles.

Supplement B: 6MWT Animations

"Supplementary Video B" contains animations of five strides in each condition of the example use case—No Intervention, FES, and AFO. These clips show the point cloud transformed into the world frame and animated in real time (unless noted). Such an animation allows researchers and clinicians to revisit data collections to visually detect any abnormalities or note any interesting gait patterns.

Supplement C: Identification of a stumble via whole foot reconstruction

"Supplementary Video C" is an animation of a person's foot during a stumble as reconstructed by the proposed method. This stumble was detected as a case when the mFC was abnormally negative. Recall, this method transforms the shoe's point cloud into the world frame as a rigid body. As such, as the heel lifts at the end of stance phase, the toe joint does not bend and the toes appear to go through the floor.

Supplement D: Minimum foot clearance during different walking styles

The figures below provide additional context to further understand the different walking styles and corresponding trajectory and acceleration outcomes. “Supplementary Video D” contains recordings of each style.

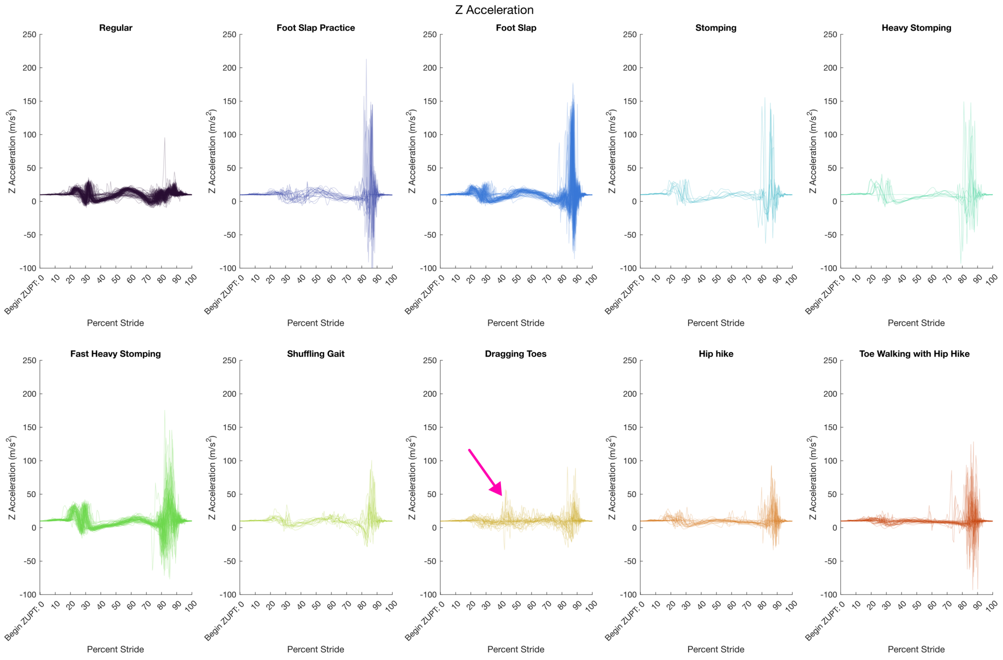

Supplemental Figure D-1. Right foot Z acceleration (global vertical acceleration) when imitating different walking styles. Each thin trace represents a single stride. Pink arrow (dragging toes) indicates the extra acceleration peak that results from the toes hitting the ground. It may also contribute error to the IMU reconstruction.

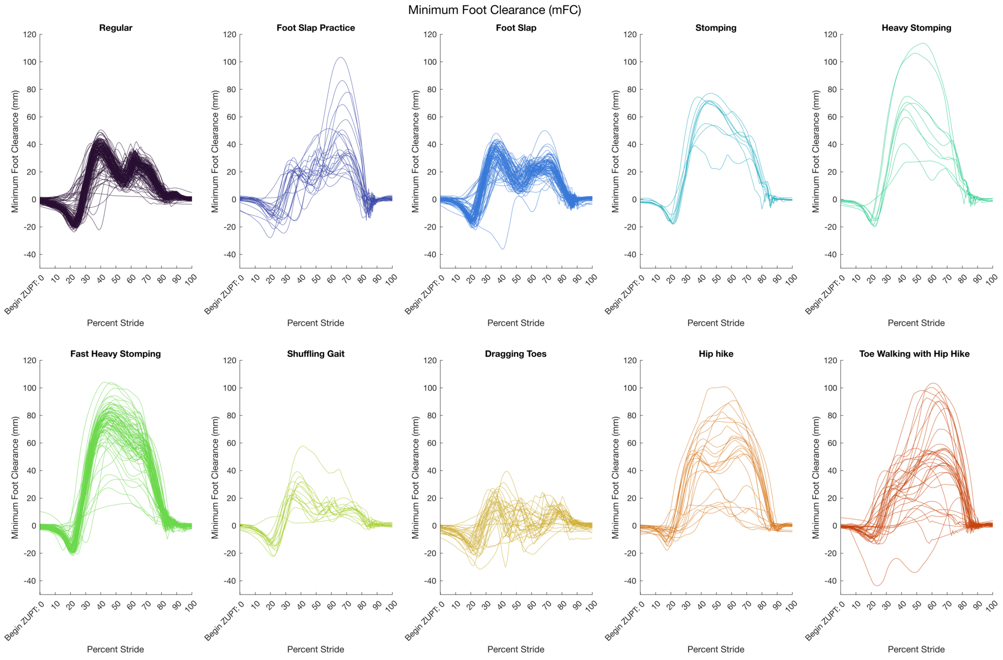

Supplemental Figure D-2. Right side minimum foot clearance (mFC) when imitating different walking styles. Each thin trace represents a single stride. In each case, the earliest dip of the curve below zero is from the toe

section of the foot during push-off, while the foot is still on the ground – the reconstruction does not bend the toes as the real foot does, so the reconstructed toes protrude into the ground. In most cases shown, there is an inflection in the middle of the mFC curve as the lowest point moves from the forefoot to the hindfoot.

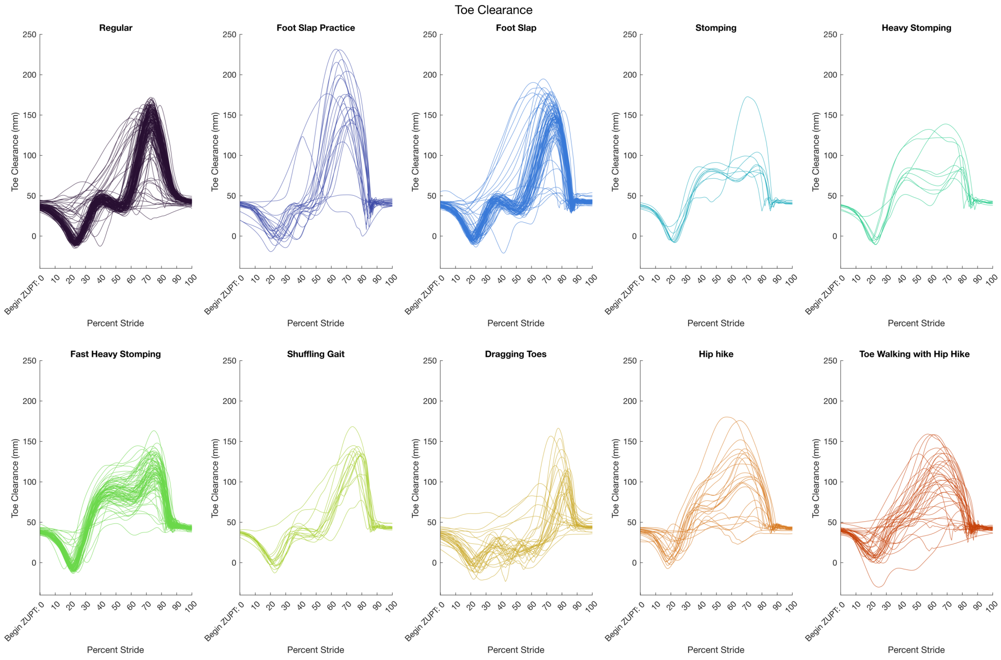

Supplemental Figure D-3. Right toe clearance when imitating different walking styles. Each thin trace represents a single stride. The toe is normally lower than the heel during the early part of swing. As above, the early dip is from the reconstructed toes protruding into the ground during push-off.

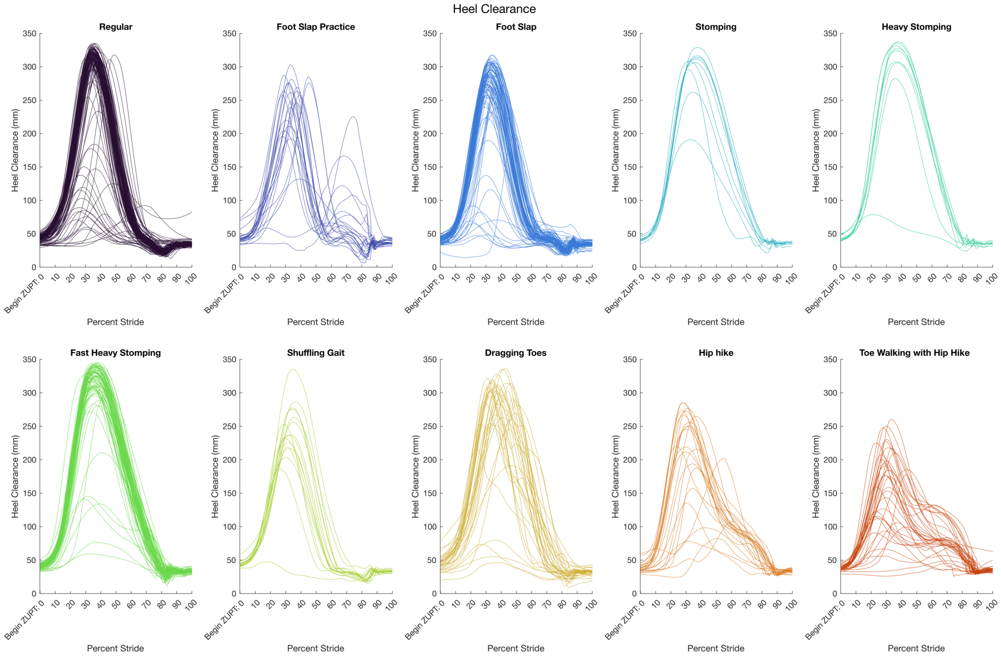

Supplemental Figure D-4. Right heel clearance when imitating different walking styles. Each thin trace represents a single stride. The heel is normally lower than the toe during the latter portion of swing.

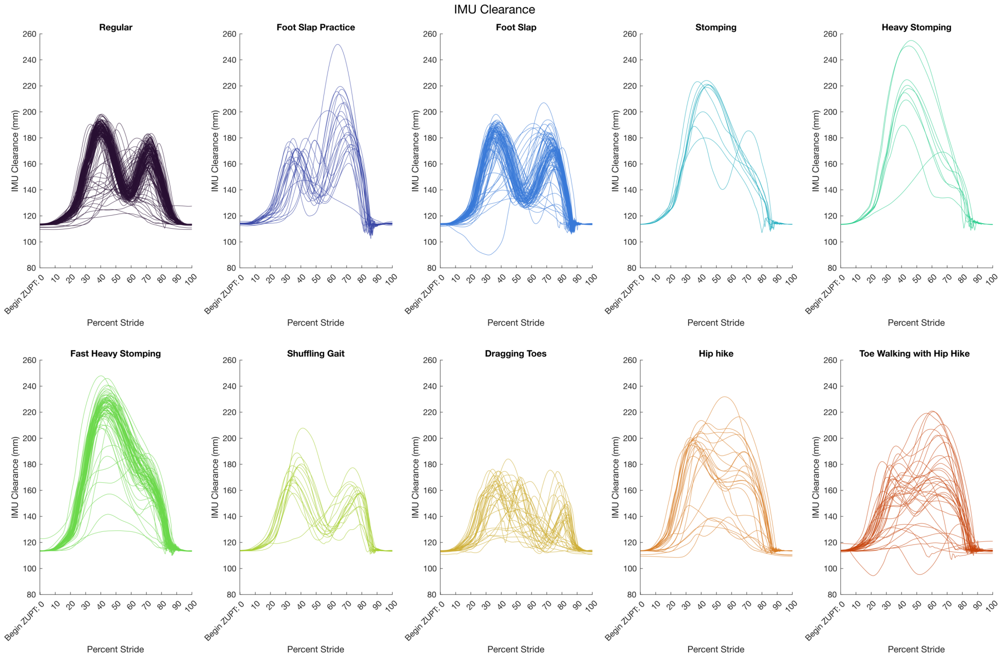

Supplemental Figure D-5. Right foot IMU clearance when imitating different walking styles. Each thin trace represents a single stride. IMU virtual clearance is one way to estimate foot clearance – the height of the middle dip of this curve above the line connecting starting and ending (stance phase) heights.

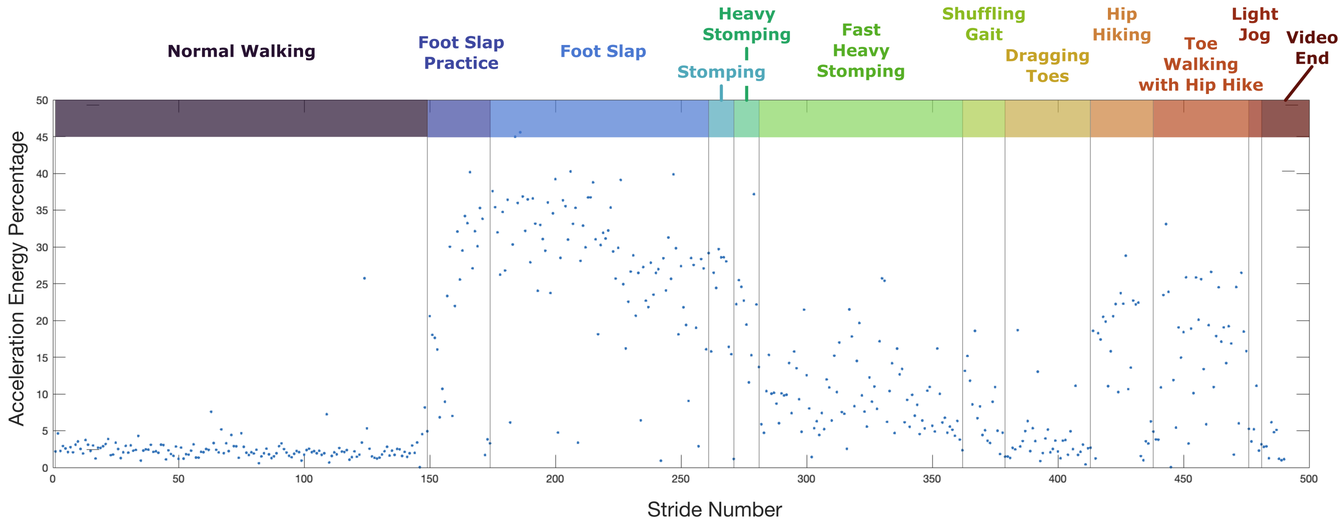

Supplemental Figure D-6. High-frequency acceleration energy percentage in each stride, when imitating different walking styles. A potential source of error in the IMU reconstruction is the large spike in acceleration at heel strike. We generated this chart to understand which styles of walking had greater high-frequency acceleration energy and determine if this could be related to a negative clearance. High-Frequency Acceleration Energy Percentage is determined by computing the power spectrum density of the raw acceleration magnitude, integrating it from 40 to 64 Hz, and finally dividing by the integral from 0 to 64 Hz (total acceleration energy for a 128 Hz sampled signal).
